# Design of percutaneous transluminal coronary angioplasty balloon catheters

**DOI:** 10.1186/s12938-023-01155-2

**Published:** 2023-09-23

**Authors:** C. Amstutz, J. Behr, S. Krebs, A. Haeberlin, R. Vogel, A. Zurbuchen, J. Burger

**Affiliations:** 1 School of Biomedical and Precision Engineering, University of Bern, Güterstrasse 24/26, CH-3008 Bern, Switzerland; 2 SMD Swiss Medical Devices, Beringen, Switzerland; 3 Department of Cardiology, Inselspital, Bern University Hospital, University of Bern, Bern, Switzerland; 4 Department of Cardiology, Buergerspital Solothurn, Solothurn, Switzerland

**Keywords:** PTCA balloon catheter, Medical devices, Catheter design, Balloon design, RX-Port, Hypotube

## Abstract

**Background:**

Eight commercially available percutaneous transluminal coronary angioplasty (PTCA), including semi-compliant and non-compliant balloons, have been assessed in detail on their tip, balloon, shaft, RX-Port, and hypotube design. Important performance characteristics such as tip deformation, balloon elongation, and deflation rate have been quantified.

**Methods:**

Five catheters of each model were evaluated during various tests. The robustness of the tips was evaluated through compression, measuring any occurrence of damage. The longitudinal growth of the balloons was recorded during inflation up to Rated Burst Pressure (RBP). The forces required to move the catheter forward and retract it into the guide catheter were measured in a simulated use test setup. The deflation behavior was studied by measuring extracted contrast media over time. Furthermore, balloon compliance and catheter dimensions were investigated.

**Results:**

The outer dimensions of the catheter were found to be smallest at the hypotube (0.59–0.69 mm) and highest at the balloon, respectively, the crossing profile (0.9–1.2 mm). The tip diameter increased after compression by 1.7–22%. Cross-sections of the folded balloons revealed a tri- and two-fold, respectively. The measured balloon elongation ranged from 0.6 to 2.0 mm. After the inflation of the balloon, an increase in friction between the guide wire and the catheter was observed on four catheters. A maximum increase of 0.12 N to 1.07 N was found. Cross-sections of the RX-Port revealed a semicircular-shaped inflation lumen and a circular guide wire lumen. The measured deflation rate ranged from 0.004 to 0.013 µL/s, resulting in an estimated balloon deflation time of 10.2–28.1 s.

**Conclusion:**

This study provides valuable insights into the design characteristics of RX PTCA balloon catheters, which can contribute to facilitating the development of improved catheter designs and enhancing clinical outcomes. Distinctions between SC and NC catheters, such as balloon performance and dimensions, are evident. It is important to note that no single catheter excels in all aspects, as each possesses unique strengths. Therefore, it is essential to consider individual intervention requirements when selecting a catheter.

The research also identifies specific catheter weaknesses, such as reduced wall thickness, fringes at the tip, and reduced performance characteristics.

## Background

Percutaneous Transluminal Coronary Angioplasty (PTCA) is a minimally invasive procedure to treat stenoses or total occlusions of coronary arteries. The procedure involves using a catheter with a balloon on the distal end. The balloon is delivered to the diseased coronary artery through a small puncture, typically made in the groin or wrist through the radial or femoral artery, respectively. The balloon is advanced through the aorta into the coronary artery until it reaches the target lesion, using a Guiding Catheter (GC) and a Guide Wire (GW). Once in a position, the balloon is inflated to dilate the diseased vessel to restore blood flow [1].

Since Andreas Grüntzig successfully performed the first PTCA in 1977 [2], there have been numerous advancements in percutaneous coronary interventions (PCI) [3–5] and the general understanding of coronary artery disease [6–8].

The initial PTCA balloon catheters from 1977 underwent various development steps. The first balloons for PTCA were manufactured from soft materials like flexible polyvinyl chloride (PVC) [2]. Nevertheless, the necessity for thick walls resulted in the development of large-profile balloons [3], posing a challenge when delivering them to the target lesion. By introducing new materials like polyethylene terephthalate (PET) and nylon [3], the size and profile of PTCA balloons have decreased, allowing improved lesion crossing, more precise placement, and maneuverability within the vessel. Besides the diameter and wall thickness (WT), the choice of material also influences the compliance of the balloons. Soft materials like low-density copolymer polyether block amide (PEBAX) [9] are used for semi-compliant (SC) balloons, while non-compliant (NC) balloons are made from nylon or PET.

The soft material makes the SC balloons allow for easy advancement through tortuous vessels. However, they come with lower Rated Burst Pressures (RBP) and tend to show the so-called dog-bone effect [9]. The latter manifests itself in a non-uniform balloon dilatation upon inflation, which manifests in a lower radial expansion at the stiffer sites of the stenosis. Non-uniform dilation risks damage to the vessel or inferior clinical outcome due to incomplete dilatation. The NC balloons are much less prone to this effect. They offer only minimal growth in diameter during the inflation and can be used until higher RBP, for example, for calcified lesions. However, sometimes even a higher RBP than the NC balloons offer is required to treat heavily calcified lesions. Therefore, manufacturers try to build catheters that sustain even higher pressures. In 2012, the first super high-pressure (> 30–45 atm) PTCA balloon catheter was brought to the market [10–12].

**Fig. 1:**
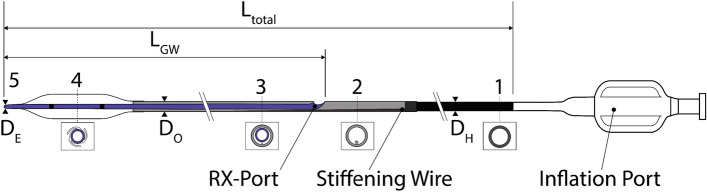
General scheme of an RX PTCA Balloon Catheter. 1: metallic hypotube, 2: transition between proximal and distal part, 3: distal part of the catheter including the inner shaft for the guidewire and the outer shaft for inflating the balloon, 4: (folded) balloon with radio-opaque markers, and 5: tip

In 1982, over-the-wire technology was introduced [13]. This technology allows a GW to pass through the entire catheter, be independently manipulated [3], and allows the physician to leave the GW in place even when exchanging the balloon catheter. However, two operators were required due to the required length of the GW (approximately 300 cm), resulting in longer treatment durations and the risk of contamination of the GW. In 1985, Rapid Exchange (RX) catheters (see Fig. 1) were introduced to overcome these disadvantages. Since then, they have become the most significant market share due to the easier handling and shorter treatment duration.

Figure 1 shows the general overview of an RX PTCA balloon catheter. The balloon catheter is inflated via Inflation Port on the proximal side of the catheter. The Inflation Port is followed by a metallic hypotube (1) with an outer diameter (OD) ${D}_{H}$. A stiffening wire (2) with a taper in the distal direction steadily reduces the stiffness of the hypotube. Besides this, the stiffening wire crosses the RX-Port and acts as a kink protection. The RX-Port is used as an exit for the GW. The distal part of the catheter (3) combines the Outer Shaft (OS) for the inflation media with the OD ${D}_{O}$, and the Inner Shaft (IS) with a length of ${L}_{GW}$ for the GW. Before the inflation of the balloon (4), it is folded to have a minimal diameter ${D}_{B}$ to facilitate the crossing of the lesion. Radio-opaque markers are applied to the inner shaft inside the balloon for correct placement. The tip (5) defines the lesion entry (LE) diameter ${D}_{E}$ and marks distal the end of the PTCA balloon catheter.

Even though balloon catheters have existed for several decades, not much literature about their design is publicly available [4, 9, 14, 15]. Furthermore, in the past, Barkholt et al. [16] investigated the tip design of PTCA balloon catheters. Gupta [17] explained PTCA balloon 'catheters' general design and properties [18]. Stent systems and the interactions with the balloon have been extensively studied and simulated [19–27]. Manufacturers provide some general but very limited information about their catheter design.

Knowledge about design properties may help to facilitate further development in this area and potentially reduce catheter costs and periprocedural risks [28–32]. For this study, eight commercially available state-of-the-art PTCA catheters have been selected based on innovative designs, market research, and interviews with interventional cardiologists and tested regarding their design of the tip, the RX-Port, the hypotube, and the dimensions of the catheter part. Furthermore, the deflation characteristics of the catheters are studied. Moreover, the balloon elongation during inflation and a potential increase in pull-back forces of the catheter along the GW and into the GC have been investigated.

## Results

### Tip design

**Fig. 2:**
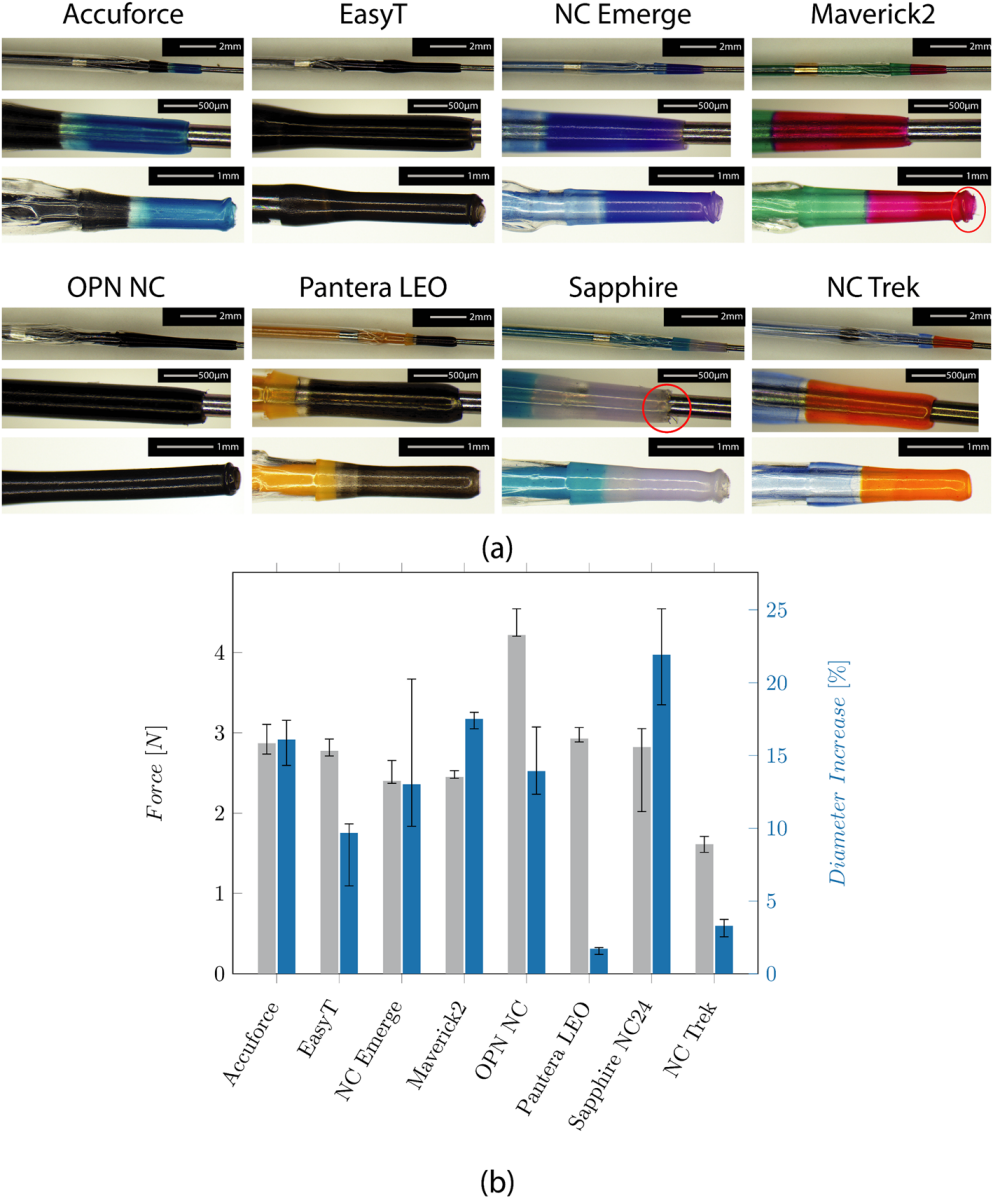
**a** Microscopic images show the folded balloon with the distal radio-opaque marker (upper row), the tip design (mid row), and the damaged tip after testing (bottom row) of the catheters. **b** Median forces and Inter Quartile Range (IQR) required to compress the tip by 0.5 mm, and the diameter increase of the tips after testing

Figure 2a shows the microscopic images of the individual tip designs. All but the NC Trek catheter, which seems to use filled polymers, have metallic (platinum-iridium) radio-opaque markers. The ones at the NC Trek seem to be a filled polymer**.**

The tapered tip is manufactured from an extra part indicated by a different color for most catheters. No color transition is visible for the EasyT and the OPN NC, and the tip shows a constant diameter before increasing at the distal balloon weld. Indicated by a red circle, fringes are visible on the tip of the Sapphire NC24 and the Maverick2.

The required forces to compress the tip and the diameter increase caused by the wrinkling of the tip can be seen in Fig. 2b. They range from 1.6 (NC Trek) to 4.2N (OPN NC). The lowest increase was found at the Pantera LEO (1.7%) and the NCTrek (3.3%). The highest increase was found at the Sapphire NC24 (22%). The tip force was highest at the OPN NC and lowest on the NC Trek.

### Balloon design and balloon elongation

**Fig. 3:**
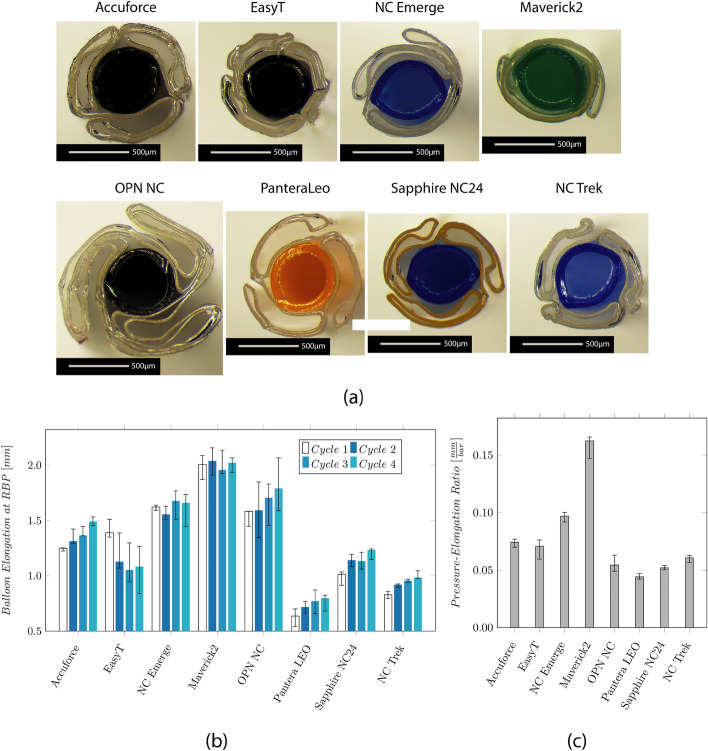
Results of the investigation on the balloon design and longitudinal elongation measurement. **a** Microscopic images show the cross-section through the center of the balloon. The balloon folds around the IS, and the wall thickness (WT) of the balloon and IS are visible. **b** Median values and IQR of the axial balloon elongation at the respective RBP in a water bath at 37 °C. **c** Median values and IQR of the ratio between the longitudinal elongation and the RBP

The folding of the balloon is shown in Fig. 3. All catheters showed a tri-folded design except the NC Emerge and the Maverick2. For the NC Emerge and the Maverick2, a two-folded design is used. The NC Emerge shows a non-symmetrical folding with a larger and smaller flap. The cross-section of the OPN NC further shows the twin-layered balloon design.

The longitudinal balloon elongation for each cycle is depicted in Fig. 3b. The measured elongation ranges from 0.6 mm (Pantera LEO) to 2.0 mm (Maverick2). It increases over the four measurement cycles for the Accuforce, OPN, Pantera LEO, Sapphire NC24, and NC Trek. The ratio between the elongation and the RBP is depicted in Fig. 3c. Low values mean a slight increase, while high values mean a significant increase when pressure increases.

Polymers tend to show an increasing strain when put under constant load. This effect, called creeping, becomes visible after reaching the RBP when the balloon further grows in the longitudinal direction. The creep distance lies between 0.14 and 0.46 mm, approximately 10–31% of the balloon elongation. The values are depicted in the Appendix in Fig. 10. The creeping effect reduces when the inflation is repeated multiple times.

**Fig. 4:**
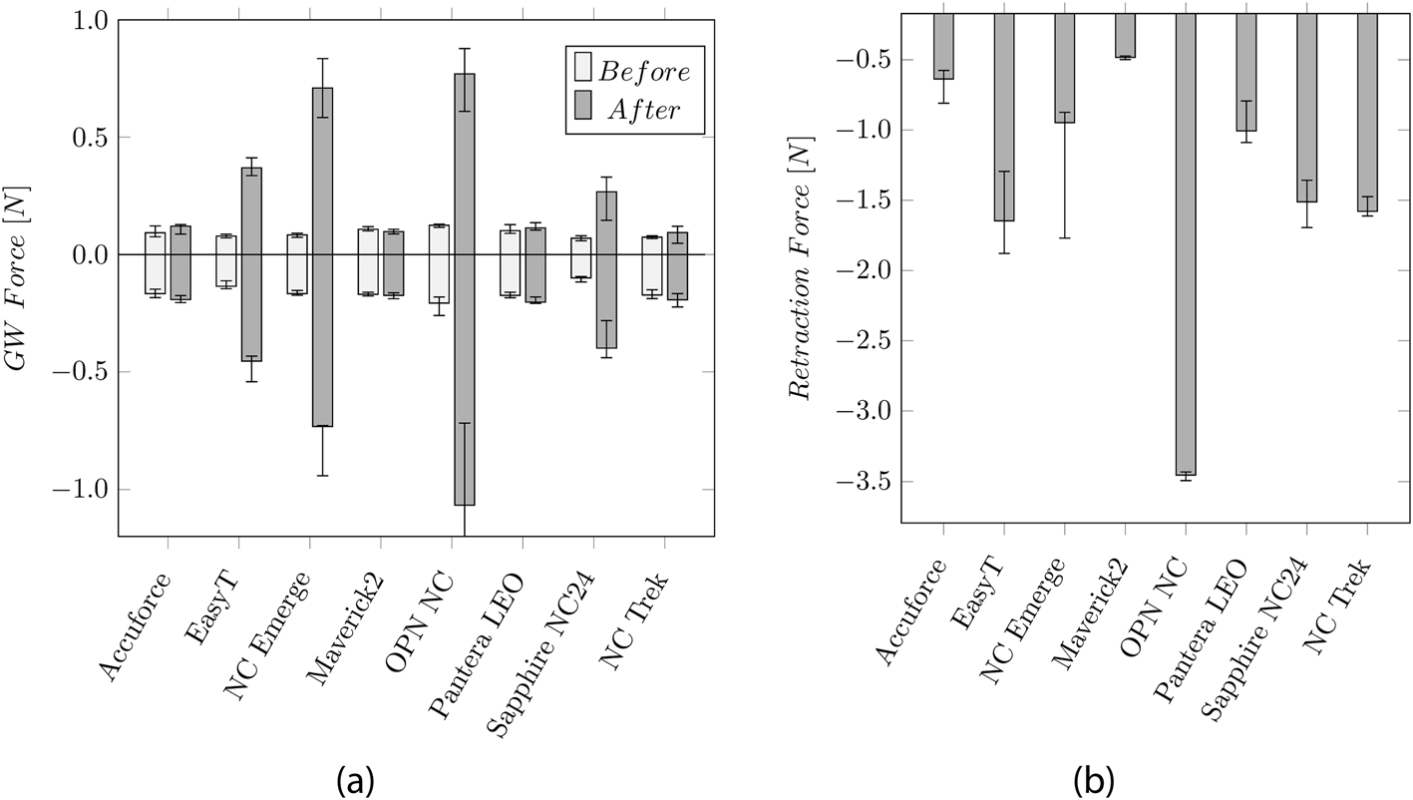
**a** Median values and Inter Quartile Range (IQR) of the GW push and pull forces before and after the inflation cycles. **b** Median values and IQR of the retraction force of the balloon into the GC

The measurement of the GW forces with the hand-held catheter force measurement device (CFMD) before and after the inflation cycles are shown in Fig. 4a. After the inflation cycle, the required push and pull forces increase from approximately 0.12 and 0.21 N to 0.37 and − 0.46 N (EasyT), ± 0.73 N (NC Emerge), 0.76 and − 1.07 N (OPN NC), and 0.26 and − 0.4 N(Sapphire NC24). The highest forces were measured on the OPN NC.

The forces, when retracting the balloon into the GC, are shown in Fig. 4b, ranging from 0.49 N (Maverick2) to 3.45 N (OPN NC).

The radial balloon growth range lies between 3% (Sapphire NC24) and 12% (Maverick, OPN). The NC and SC balloons show compliances between 0.06 and 0.09 mm2/atm and 0.19–0.23 mm2/atm, respectively.

The compliance charts based on the manufacturer's data are in the Appendix (cf. Fig. 11a). The resulting balloon growth and compliance based on these charts are shown in Fig. 11b.

### Shaft design and dimensions

**Fig. 5:**
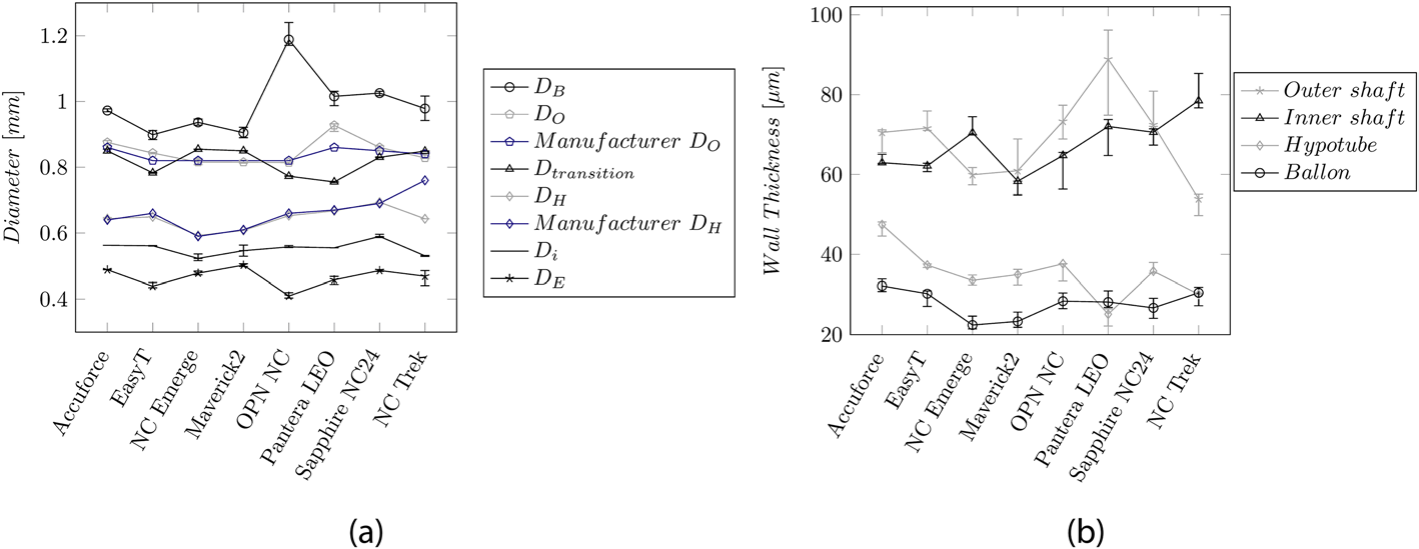
**a** Median values and IQR of the measured diameters along the catheter length. ${D}_{B}$: Balloon, ${D}_{O}$: OS, ${D}_{H}$: Hypotube, ${D}_{i}$: IS, ${D}_{E}$: Entry profile. The blue curves indicate the dimensions provided by the manufacturers. **b** Median wall-thickness and IQR for the OS, IS, and balloon

Figure 5a, b depicts the diameter of the catheter sections and the WT, respectively. The LE profile at the tip ${D}_{E}$ shows the smallest diameter (0.40–0.50 mm). The highest diameter (0.9–1.2 mm) was found on the balloon. This value defines the crossing profile (CP) and is typically indicated by the maximum diameter between the proximal balloon weld and the tip, usually located at the proximal end of the balloon.

The diameter ${D}_{O}$ of the OS (0.81–0.93 mm) and the shaft at the transition between the proximal and distal part (0.75–0.85 mm) are comparable. The diameters ${D}_{i}$ of the IS and the hypotube ${D}_{H}$ were between 0.53–0.56 mm and 0.59–0.69 mm, respectively.

The WT of the OS, IS, and hypotube vary between the manufacturers in a range of 54–89 µm, 58–78 µm, and 25–47 µm, respectively. The balloon's stretch-blow-molding manufacturing process allows for small WT (22–32 µm).

### RX-Port design and deflation rate

**Fig. 6:**
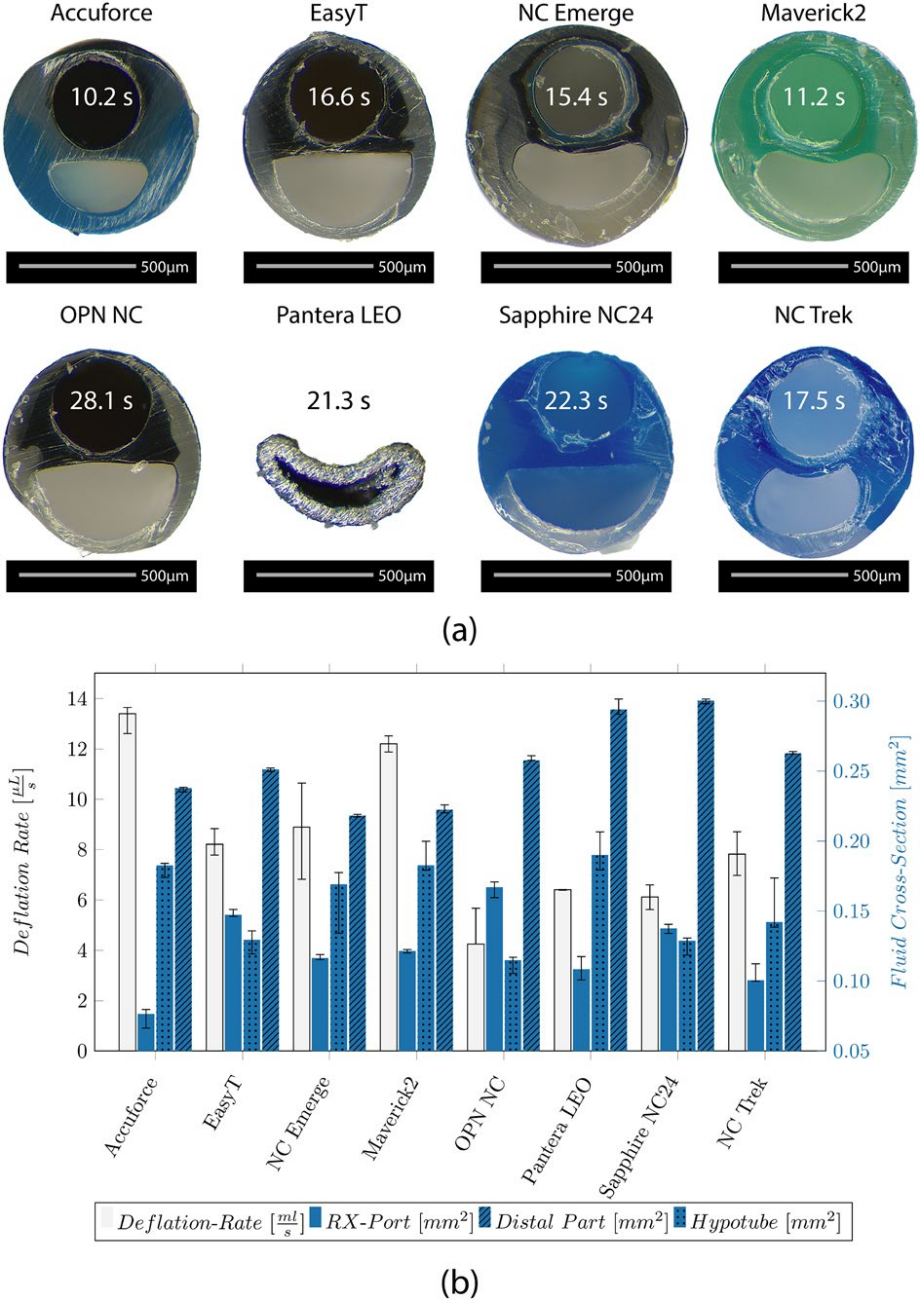
**a** Microscopic image of the cross-section of the RX-Port showing the GW lumen and the fluid cross-section and median value of the deflation rate in comparison to the median value of the deflation time for a balloon with a diameter of 3 mm and a length of 20 mm. **b** Median values and IQR of the deflation rate compared to the median values and IQR of the fluid cross-section located at the RX-Port, the distal part, and the hypotube

Figure 6a shows the cross-section of the RX-Port design for the different catheters. Notably, catheters from the same manufacturer, like the EasyT and the OPN NC, and the NC Emerge and the Maverick2, exhibit a similar fluid shape. The EasyT and the OPN NC demonstrate the largest fluid cross-section among the group. For catheters like the NC Emerge, Maverick2, and the NC Trek, it becomes visible from the cross-section that a multilayer IS is used.

The measured deflation rate is shown in Fig. 6b. Furthermore, the fluid cross-section at the RX-Port, the distal part, and the hypotube are evaluated. Generating cross-sections of the Pantera LEO was challenging due to the metallic RX-Port, and resulted in deforming the shape. Therefore, the fluid cross-section was estimated based on the diameter and the WT.

The fastest deflation was observed for the Accuforce (0.013 µL/s) and the Maverick2 (0.012 µL/s), and the slowest for the OPN NC (0.004 µL/s). The calculated median deflation time for a balloon with a diameter of 3 mm and a length of 20 mm is indicated on the respective catheter cross-section in Fig. 6a.

### Hypotube design

**Fig. 7:**
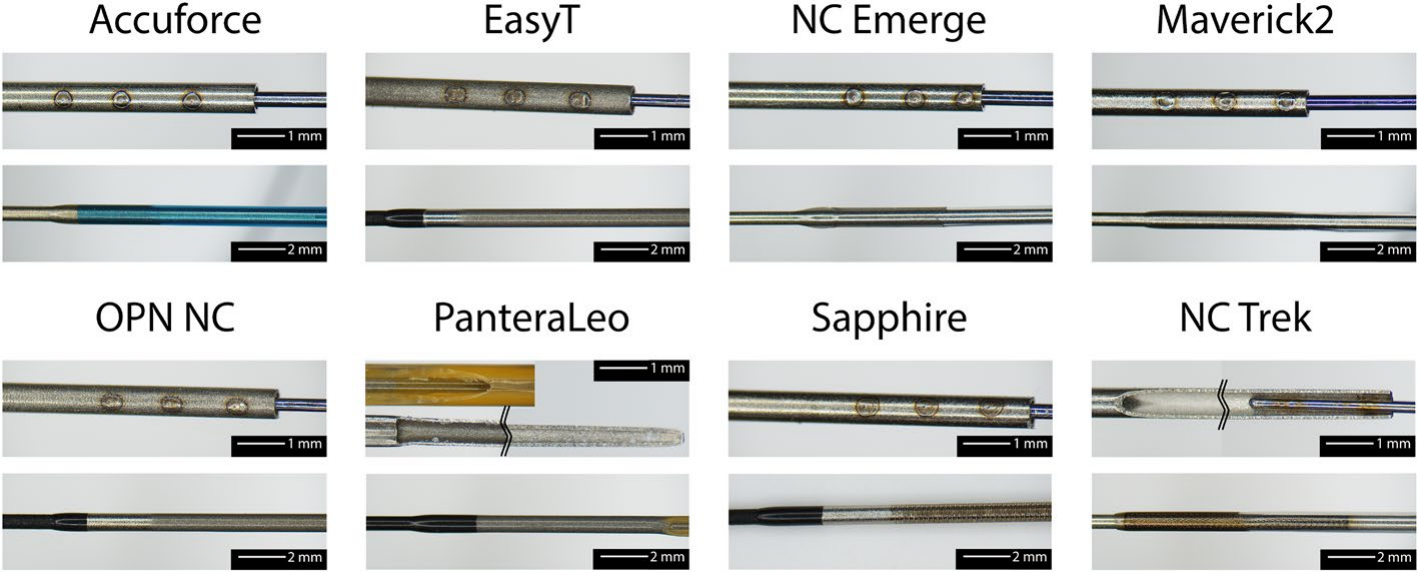
Microscopic image of the transition between the hypotube to the distal part. Attachment of the stiffening wire (upper row) and attachment of the OS to the hypotube (bottom row)

The design of the transition between the hypotube and the distal part is shown in Fig. 7. Most catheters, except the Pantera LEO and the NC Trek, show a three-spot welding of the tapered stiffening wire to the hypotube. The hypotube of the Pantera LEO is distally reshaped and skived, and the RX-Port is located directly at the end of the hypotube. Additionally to the stiffening wire, the hypotube of the NC Trek is skived distally. At the location where the OS is attached to the hypotube, a roughening of the hypotube's surface is visible for most catheters. The OD and WT of the hypotube are depicted in Fig. 5a, b, respectively.

The measured lengths of the catheter sections (transition length, GW length, and the length of the stiffening wire) are depicted in the Appendix (cf. Fig. 12a). The transition length indicates the distance between the end of the hypotube to the RX-Port and ranges from 71 to 115 mm. The Pantera LEO does not show this transition since the RX-Port is located directly at the end of a skived hypotube. The GW length indicates the supported GW length and runs from the RX-Port to the tip. Except for the Pantera LEO, this length lies between 230 and 250 mm. For all catheters, the stiffening wire (33–265 mm) is longer than the transition length, indicating that the wire overlaps the RX-Port by 30–180 mm and even extends to the balloon for the Sapphire NC24. Furthermore, Fig. 12b) in the Appendix depicts the base diameters at the hypotube (25–34 µm) and the tip diameter (8–13 µm) of the stiffening wire.

## Discussion

In Table 1, an overview of the measurements is shown. The measured values are classified from '–’ (least favorable) to ‘ +  + ’ (most favorable). This overview shows the strengths and weaknesses of the design elements of the investigated catheters compared to each other. It must be noted that all tested catheter types (SC and NC) were included during the normalization. Even though SC and NC balloon catheters are different types and usually not comparable, it allows showing differences between these groups.

**Table 1:** Linear normalized rating of the individual catheter features

Category	Accuforce	EasyT	NC Emerge	Maverick2	OPN NC	Pantera LEO	Sapphire NC24	NC Trek
LE	–	+	–	–	+ +	0	–	–
Tip increase	–	0	0	–	0	+ +	–	+ +
Tip force	0	0	–	–	+ +	0	0	–
RBP	0	–	–	–	+ +	–	0	–
Radial Growth	+	–	+ +	–	–	+ +	+ +	+
Compliance	+	-	+ +	–	+ +	+ +	+ +	+
Elongation	0	+	–	–	–	+ +	+	+
GW Clamping	+ +	0	–	+ +	–	+ +	–	+ +
Pull-out	–	0	–	–	+ +	–	–	–
CP	+	+ +	+	+ +	–	0	0	+
Ø OS	0	+	+ +	+ +	+ +	–	0	+
Ø Hypotube	0	0	+ +	+	0	–	–	0
Deflation Rate	+ +	0	0	+	–	–	-	0

Normalized values were classified from—(least favorable) to +  + (most favorable)—Black: NC balloons, Blue: SC balloons, Orange: Super high-pressure NC balloons

From this overview, it can be seen that the SC Maverick2 shows a reduced tip and balloon performance when compared to the NC balloon catheters. However, the dimensions are small, enabling, for example, the treatment of non-calcified stenoses in very tortuous vessels. Both SC catheters, EasyT and Maverick2, exhibit excellent CPs. Even though the EasyT shows higher dimensions, the tip and balloon performance is slightly superior to the Maverick2.

Comparing the NC balloons against each other shows that the Pantera LEO, the Sapphire NC24, and the NC Trek show excellent balloon performance. However, the Pantera LEO might have some drawbacks regarding dimensions and deflation rate. The OPN NC has excellent tip performance and unique features, like high RBP and low compliance required for highly calcified lesions. However, the high CP might reduce the applicability since certain stenoses cannot be crossed. Furthermore, the deflation rate is reduced.

No final conclusions in respect of catheter performance can be reached yet. In the future, parameters like pushability (the ability to advance the catheter), trackability (the ability to follow tortuous vessel paths), frictional properties, and stiffnesses of the catheter parts need to be investigated. Furthermore, it is important to note that the measured values are not directly applicable to clinical practice. The operator’s proficiency, strategy, and approach play a significant role in the execution of procedures. Additionally, the combination of the guiding catheter, guidewire, and balloon is essential. The selection and interaction of these components significantly impact the overall performance and success of the intervention.

In the following, the individual design elements and potential differences between the used test setup and the reality are discussed in more detail.

### Tip design

The tip design of the NC Trek, NC Emerge, and Accuforce was studied by Barkholt et al. [16]. It was found that the NC Trek’s design was resistant to damage. This could be confirmed in this study. Generally, the tip should be smallest distally to ensure a small lesion entry profile. However, a too-thin WT can lead to a damaged tip and the so-called *fish-mouthing effect* shown in our investigation (cf. Fig. 2) and Barkholt et al. [16]. A damaged tip may increase the crossing difficulty or cause stent distortion. Overall, the tip of the Pantera LEO showed the slightest plastic deformation. Based on experience, the Pantera LEO is inferior in crossing lesions. A reason, therefore, could be the distally increasing tip diameter (see Fig. 2a).

Since the microscopic images of the catheter tips were taken before any other tests, the fringes, visible on the tip of the Sapphire NC24 and the Maverick2 (cf. Fig. 2a), midrow), are possibly caused during manufacturing. It has to be noted that the fringes on the Maverick2 were minimal and only found on one catheter. However, the fringes on the Sapphire NC24 were found on all investigated samples. These fringes could not be observed on the other investigated catheters. These fringes are expected to be undesirable since they could enlarge the LE diameter and possibly cause vessel trauma or thrombosis in the upstream part of the coronary arteries if they get detached during an intervention.

The radio-opaque markers indicate the balloon’s location inside the patient. Thick and long markers can increase the CP and the local stiffness of the balloon. Among all investigated catheters, the NC Trek is the only one that uses filled polymeric markers (tungsten-PEBAX). Due to the increased flexibility of the markers, performance characteristics like trackability might be improved. Furthermore, the welding process could increase the accuracy of the marker placement during the manufacturing process. Since these markers are usually welded but crimped, the risk of causing defects at the IS during the placement is reduced. However, thermal changes during the welding process could cause degradation of the material and a thinning of the IS. Furthermore, the tungsten-filled Pebax markers show reduced radio-opacity that negatively affects the visibility during the investigation.

### Balloon design

A tight balloon folding is preferable since the CP should be as small as possible to allow easy entry of the lesion and correctly position the balloon. Both SC catheters Maverick and EasyT show a tight two- and three-folded balloon, respectively. The NC Emerge uses a different method than the other NC balloons, resulting in a tight but asymmetric wrap. The CP of the OPN NC was the largest due to its twin-layer balloon design.

Even though the OPN is an NC balloon, the overall radial growth is high (10%). However, it has to be noted that this is due to the high RBP of 35 atm. The growth has to be considered during the intervention to prevent vessel damage.

### Balloon elongation

Low longitudinal growth is desired to avoid vessel trauma and aid the correct balloon placement. Excessive elongation may result in inflation outside the stenotic area or the stent. This, in return, can lead to vessel injuries or improper stent expansion. Besides the clinical complication, a high balloon elongation can lead to plastic deformations such as narrowing of the IS, increasing the friction between the catheter and the GW. In the worst case, this may even completely trap the GW. To avoid this effect, manufacturers tend to reduce the shoulder length of the balloon to reduce the longitudinal growth. The Maverick2 showed the highest elongation. Despite the elongation, no clamping of the GW could be observed. Even though the elongation of the Sapphire NC24 and the EasyT is low, there was still an issue with GW clamping after inflation. The GW friction after the inflation indicates plastic deformation of the IS. After inflating the OPN, the GW was almost stuck and could not be removed while retracting the catheter through the GC. It has to be noted that only one GW (ASAHI SION Blue) has been studied. Based on talks with physicians, the GW clamping of the OPN NC, for example, is reduced or vanishes for other GWs. Therefore, the plastic deformation might not be the sole trigger of this effect but also an issue with the interaction between the IS and the coating/surface of the GW.

A catheter stuck on a GW might be a serious issue during the intervention. It can negatively impact the duration and impose a risk of injury since the GW has to be replaced. The tests have only been performed after the four inflation cycles. In the future, it would be interesting to study, whether this effect already occurs after the first inflation or only happens over multiple inflations. During the bench test, the outer surface of the balloon was “free” and not in contact with any artery, which might influence the elongation. However, each catheter’s test conditions were the same, resulting in comparable values.

The retraction forces to pull the catheter back into the GC can indicate the rewrapping behavior of a balloon. However, during the intervention, the balloon is inflated inside an artery. The surrounding artery can help the catheter partially rewrap.

The highest retraction forces have been measured at the OPN NC, probably due to the twin-layered balloon. The high forces required during catheter removal must be considered in the fracture properties of the catheter shafts. On one hand, high retraction forces inside the vessel can pull the GC into the artery causing a vessel injury. On the other hand, high forces can lead to the GC being pulled out of the ostium and requiring it to be repositioned.

During the GW/catheter force measurement, the CFMD must be perpendicular to the catheter. Since the motion is carried out by hand, some influences of the operator are possible. All tests have been performed by the same person, taking care of the correct alignment. The device could further be used during interventions to give more insight into the applied forces by the interventionalist. These values are unknown and could help better understand the catheters’ performance.

### Dimensions

Small diameters, CP, and entry lesion profiles are preferable since they are easier to navigate, to cross the lesion, and enable “*Kissing techniques*” to treat bifurcations [8]. The WT of the Maverick’s OS is the thinnest. For all catheters, the variance of the balloon WT is higher than that of the shafts. This might be due to balloon fabrication [33]. A multilayer IS is visible for some catheters, like the NC Emerge, Maverick2, and the NC Trek. The inner layer of the IS is usually designed to reduce the friction between the GW and the IS.

### RX-Port design

Manufacturing the RX-Port can be done using a coated wire to stabilize the IS and a coated and shaped wire, which defines and stabilizes the shape of the fluid cross-section at the RX-Port. The difference between the used wires for the inflation lumen is visible in Fig. 6a. The EasyT and OPN NC have the largest fluid cross-section at the RX-Port. However, this results in thin WT, which in turn can negatively impact the tensile strength and RBP of the RX-Port. Rounded edges are preferable for the stress distribution in the polymer during inflation. However, manufacturing such stabilization wires for the fluid cross-section could be more complex. The hypotube design of the Pantera LEO makes manufacturing the RX-Port easier since no additional stabilization wire for the fluid cross-section is required. Additionally, this hypotube design eliminates one welding step as the hypotube and the distal part of the catheter are joined at the RX-Port. Hence, the fabrication of the RX-Port is directly realized through joining the distal part to the hypotube. The other catheters presented in this study include a separate weld several centimeters proximal to the RX-Port for attaching the transition part to the hypotube.

The deflation time is influenced by the circular cross-section at the hypotube, the circular ring cross-section between the OS and IS, and the RX-Port design. In general, it can be said that the smaller the circular ring of the hypotube and the narrower the circular ring at the distal part of the catheter, the longer the deflation time.

The calculated deflation time based on the measured deflation rate for a balloon with an OD of 3 mm and a length of 20 mm is relatively long (10–28 s). A reason can be that the 7 ml in the syringe was too much, reducing the generated vacuum, or that the contrast media—saline solution temperature was 23 °C instead of 37 °C, resulting in a higher viscosity. Furthermore, the pressure inside the coronary arteries, along with the elastic recoil of the balloon, aids in pushing the fluid out of the catheter. The deflation time was likely overestimated since neither of these two effects were present during the test. However, the conditions for all catheters were the same; therefore, the deflation rate can be used to compare the catheters.

All catheters’ guided GW (${L}_{GW}$)- and total length are comparable, except for the Pantera LEO. Its IS is more extended, resulting in a longer-guided GW length. The higher length might influence the performance of the catheter. However, further tests would be required to make conclusions. In general, it can be said that ${L}_{GW}$ should be sufficiently long to ensure that the RX-Port remains inside the GC throughout the intervention.

### Hypotube design

Due to the geometry, the RX-Port is generally more prone to kinking than the rest of the catheter. Therefore, the tapered stiffening wire crosses the RX-Port for all catheters. The stiffening wire prevents the catheter from kinking and generates a smooth transition from the highly stiff metallic hypotube toward the flexible polymeric distal part.

The stiffening wire of the Sapphire NC24 even extends until the balloon. Since the wire is thin, no significant influence on the bending stiffness is expected (approximately 1–2%). However, an extended wire can improve the overall kink resistance and the transmitted force to the tip.

The most common design involves a three-spot weld of a separate tapered wire onto the hypotube. The Pantera LEO presents an alternative design approach. Instead of using a separate tapered wire, the hypotube was reshaped into a half-moon and skived to reduce the stiffness towards the distal end. As stated above, this approach makes RX-Port welding easier since it eliminates the need for an additional stabilization wire for the fluid cross-section. However, manufacturing of a hypotube as for the Pantera LEO might involve more steps due to its more sophisticated design than the otherwise common three-spot weld. Moreover, during the investigations, it was observed that the skived part is more susceptible to damage during insertion than the tapered wires. While the skive at the hypotube of the NC Trek does not reduce distally, it does contribute to a further reduction in the stiffness gradient from the hypotube to the distal part. This design modification simplifies the spot welding of the stiffening wire; nonetheless, it also adds to the overall costs of the hypotube due to the additional process.

Most catheters showed a roughening of the surface at the distal end of the hypotube. Since metal and polymer do not form a weld, the roughening of the surface increases the tight fit between the OS and the hypotube.

## Conclusion

In conclusion, this paper offers a comprehensive overview of the SC and NC catheters’ design elements and performance characteristics. By comparing their performance, distinctions between the two types, such as balloon performance and dimensions, become evident. It is important to note that no single catheter excels in all aspects, as each possesses unique strengths. Additionally, the interventionalist’s preferences and materials influence catheter performance. It can be concluded that balloons with high performance tend to have larger overall dimensions. Therefore, the selection of a catheter should be based on individual intervention requirements.

Moreover, this research identifies specific weaknesses in individual catheters, including reduced wall thickness at the RX-Port, fringes at the tip, potential cost reductions, and weaknesses in the tested performance characteristics. These insights contribute to a better understanding of catheter design and can facilitate the development of improved designs that enhance clinical outcomes in percutaneous coronary interventions.

## Methods

The selection of PTCA balloon catheters for this investigation was based on a comprehensive evaluation that considered unique features such as the RX-Port design or the RBP, as well as market research and interviews with interventional cardiologists. During selection, catheters with different designs and characteristics, such as super high-pressure NC, high-pressure NC, and SC, were chosen to find potential differences. A commonly used balloon size with a balloon diameter of 3 mm and a length of 20 mm was chosen for this study. However, the Sapphire NC24 was only available with a length of 18 mm. Table 2 lists the eight selected PTCA balloon catheters in alphabetical order.

**Table 2:** Overview of the analyzed catheters

Manufacturer	Balloon Catheter	Compliance	RBP [atm]	Selection criteria
Terumo, Tokyo, Japan	Accuforce	NC	22	Recommended
SIS Medical AG, Frauenfeld, Switzerland	EasyT	SC	21	SC with high RBP
Boston Scientific, Massachusetts, USA	NC Emerge	NC	20	Recommended
Boston Scientific, Massachusetts, USA	Maverick2	SC	14	Recommended SC Catheter
SIS Medical AG, Frauenfeld, Switzerland	OPN NC	NC	35	Highest RBP
Biotronik, Berlin, Germany	Pantera LEO	NC	20	Innovative RX-Port Design
Orbus Neich, Hong Kong	Sapphire NC24	NC	24	Highest RBP with single-wall balloon
Abbott, Illinois, USA	NC Trek	NC	18	Recommended

In total, five catheters of each model were used during the investigation. The median and Inter Quartile Range (IQR) were evaluated for each measured value. A GW is a rail for the tools required to treat a lesion during the intervention. Furthermore, a GC is a hollow tube that goes from the incision site to the ostium of the coronary artery to be treated. For realistic investigations, the straight GW ASAHI SION blue (ASAHI INTECC CO. LTD., Tokyo, Japan) and the 5F JL 4.0 GC (Medtronic, Dublin, Ireland) were selected based on talks with physicians for this study.

The catheter dimensions, tip design, balloon folding, elongation, RX-Port design, deflation time, hypotube design, and distal components’ length were investigated.

### Dimensions

The OD of the catheters was measured on five catheters per model, with a Keyence LS-9030D High-Accuracy CMOS Micrometer (KEYENCE International, Mechelen, Belgium) (measurement accuracy ± 2 µm). The OD has been measured at the five locations (1–5) depicted in Fig. 1. The catheter was cut into segments using the 1401 cutting device (BW-TEC AG, Hoeri, Switzerland) to measure the WT and ID of the balloon and the OS and IS. All inner dimensions and cross-sections were analyzed using a Nikon SMZ745T stereo Microscope (Nikon Instruments Inc., Tokyo, Japan) and a JENOPTIK GRYPHAX SUBRA (Jenoptik AG, Jena, Germany) camera. The accuracy of measurements with a stereo microscope depends on many factors like contrast, lighting, focus, calibration, and placement of the measurement on the picture. The accuracy of the measurement was estimated to be about 5%. The length of the parts was measured with a precision steel ruler (measuring accuracy according to EC accuracy class II).

### Tip design

The design was evaluated by recreating the bench test setup of Barkholt et al. [16]. With a GW in place, the tip was pressed against a plate with a hole slightly larger than the GW (0.36 mm). The tip was clamped into the universal testing machine Shimadzu AGS-X 10 kN (Shimadzu Corporation, Kyoto, Japan). The force required to compress the tip by 0.5 mm was recorded using a 200 N load cell with an accuracy of the measured value of ± 0.5% in the range of 0.4–200 N. Below 0.4 N, the accuracy of the measured value is reduced to ± 1%. While the GW can pass through the hole, the tip gets pressed against the plate. The damage of the tip was assessed by measuring the diameter before and after the test, respectively.

### Balloon design and balloon elongation

During inflation, the balloon does not only grow radially but also longitudinally. Markers were applied at the proximal balloon welding and on the tip to measure the longitudinal growth. They were recorded using a 12-megapixel camera and evaluated using GOM Correlate software (GOM Metrology, Brunswick, Germany). A GC with an inserted GW was placed inside a water bath with a temperature of 37 °C. Each catheter was mounted on the GW, and the balloon was placed approximately 10 cm outside the GC's distal end. The balloon was inflated to the RBP using plain water with a high-pressure inflation device (SIS Medical, Frauenfeld, Switzerland). After reaching the RBP, the pump is locked, and the volume inside the balloon is kept constant for approximately 10 s. It has to be noted that the balloon is filled with a mixture of contrast media and saline solution during interventions. The higher viscosity of the contrast media compared to water affects deflation time. However, it is important to clarify that deflation times are not the focus of this investigation and will be examined separately. In this particular study, the analysis focuses on static pressure, thereby neglecting the influence of fluid properties such as viscosity.

The software Labview 2019 (National Instruments Corp, Austin, Texas, USA) recorded the pressure using an SPT4213 pressure sensor (Stork Solutions Ltd, Aldermaston, United Kingdom). In total, the RBP was applied four times on each catheter. Since the catheters have different RBPs, the ratio between elongation and RBP is evaluated for a better comparison.

**Fig. 8:**
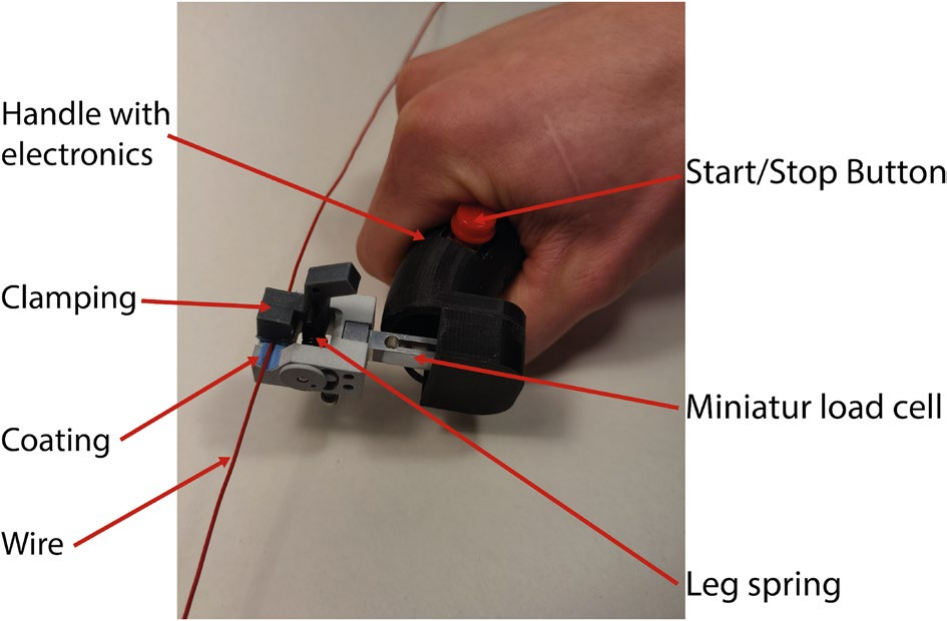
Hand-held catheter force measurement device to investigate the forces required to push or pull in a GW, GC, or hypotube. The respective device will be fixed at the clamping. By moving the handle, the clamped catheter is inserted or exserted. The required force is measured by the load cell

Longitudinal elongation, in combination with high pressures acting on the inner shaft, can cause plastic deformation. These deformations can constrict the GW with a risk of implications such as GW loss or prolonging the intervention duration. Before applying pressure, the pull and push forces required to move the GW inside the catheter were recorded CFMD (cf. Fig. 8). This device is further explained in the Appendix. After the fourth inflation, the pull and push-force measurements were repeated. In general, vascular models provide further insights into the performance of PTCA balloon catheters. However, for the measurement of the GW clamping, influences of the vascular tortuosity, balloon size, catheter bending stiffness etc., were eliminated by keeping the setup as simple as explained above. During measurements with the hand-held device, it was ensured that the device was kept perpendicular to the catheter and that a stable plateau of push and pull forces was generated for each measurement. Based on the plateau, an average force is generated. Markers on the table defined the maximum travel distance, and a clock was used to use approximately the same time for each push and pull movement. All tests have been performed on the same day by one person. The reproducibility of the measurement is tested by performing multiple push and pull cycles on one catheter (cf. Appendix Fig. 16). Finally, the balloon catheter is retracted through the 5F JL 4.0 GC, and the retraction force is recorded with the hand-held device.

Furthermore, the manufacturers’ compliance charts of the catheters were compared. Based on these charts, the balloon growth ${D}_{growth}$ (Eq. 1) and compliance (Eq. 2) were calculated between the nominal pressure ${P}_{nom}$ and the RBP, respectively.

1 $$D_{growth} = \frac{{\left( {D_{RBP} - D_{nom} } \right)}}{{D_{RBP} }}\, \cdot \,100 \left[ \% \right]$$

2 $$Compliance = \frac{\Delta D}{{\Delta P}} = \frac{{D_{RBP} - D_{nom} }}{{RBP - P_{nom} }}\left[ {\frac{mm}{{atm}}} \right]$$

### RX-Port design and deflation

After the inflation, the balloon is deflated to restore blood flow and remove the catheter. The catheter is inflated/deflated through the hub. Therefore, the inflation medium has to pass through the hypotube, cross the RX-Port, which acts as a throttle point, and the distal catheter part before it reaches the balloon. Typically, the medium flowing through the hypotube maintains a circular cross-section. However, after passing the RX-Port, the medium flows between the OS and the IS, leading to a circular ring cross-section.

**Fig. 9:**
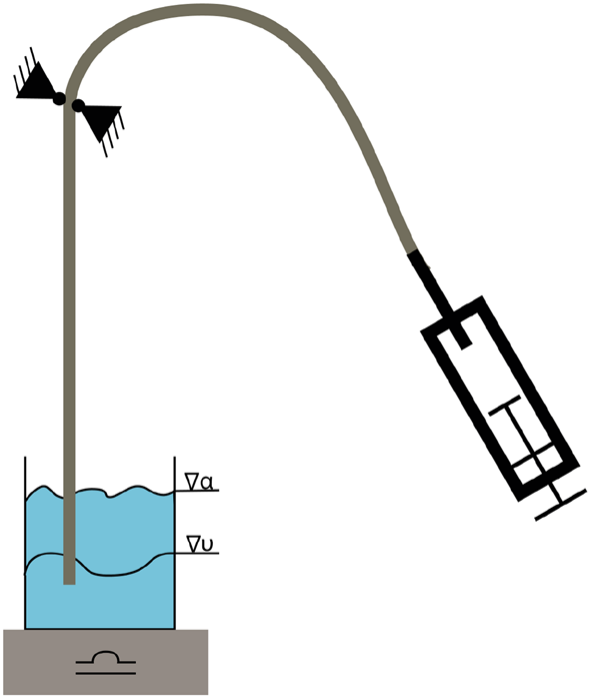
Schematic of deflation time measurement on PTCA balloon catheters. The distally opened catheter shaft is placed in 300 ml contrast media–saline solution at 23 °C and fixed in position. A syringe attached to the hypotube generates a vacuum. The water reduction is measured as a weight reduction over time

**Fig. 10:**
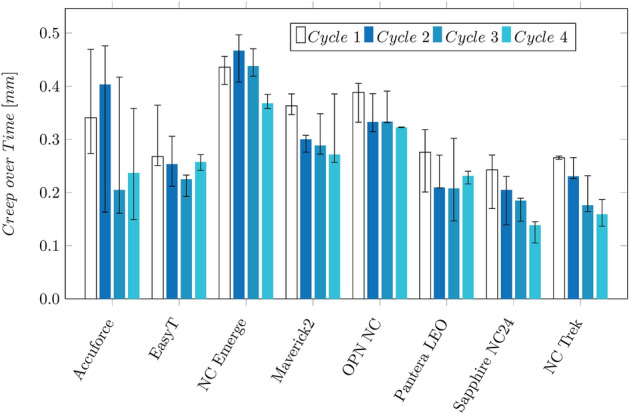
Creep during inflation time: **a** 23 °C Proximal Outershaft, **b** 37 °C RX-Port. The shaded areas indicate the scattering of the measurements (minimum and maximum values)

**Fig. 11:**
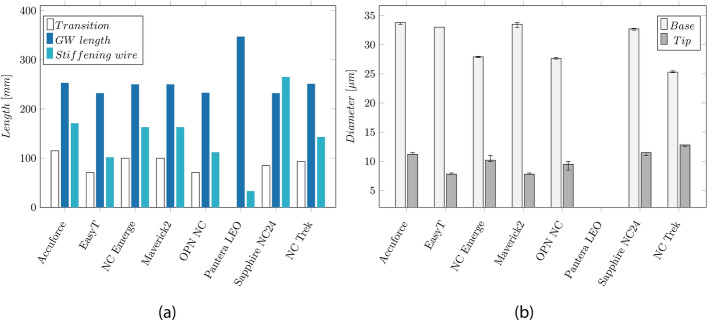
**a** Overview of the lengths of the catheter sections: Transition length, GW length, and the length of the tapered stiffening wire of the catheters. **b** Base and tip diameter of the tapered stiffening wire, whereas the small diameter is located distally of the RX-Port

**Fig. 12:**
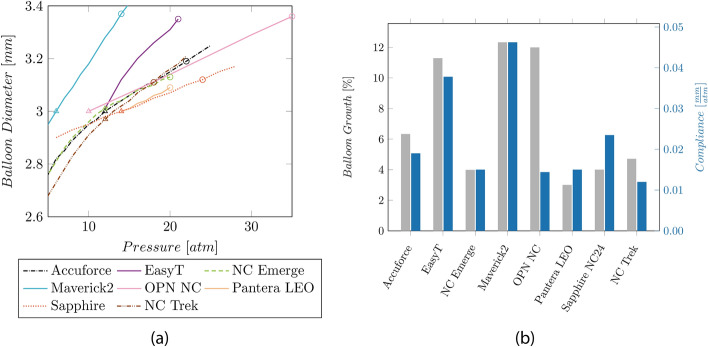
**a** Compliance chart based on the manufacturer’s data. The triangular shape indicates the nominal pressure, and the circular shape indicates the RBP. **b** Balloon growth and compliance values are based on the available manufacturer’s data.

The inflation media usually consists of a contrast agent mixed with saline solution, such as an iodine-based contrast agent like iodixanol, ioxaglate, or iopromide. The higher the viscosity of the inflation media, the longer the deflation time [34]. Because an inflated balloon prevents blood flow, a long deflation time is unpleasant for the patient. Since the contrast agent’s viscosity is generally higher than water, it was mixed with a physiological saline solution in a ratio of 1:1. The same ratio was used during this study. Furthermore, Iomeron 350 mg/ml (Bracco, Milan, Italy) was used as a contrast agent. The setup is shown in Fig. 9, and the measurement is conducted at 23 °C. The balloon was removed from the catheter for this test to obtain an open system where fluid can flow through. Prior to the test, a container was filled with 300 ml (358.17 g) of the contrast media–saline solution. Furthermore, it was ensured that the whole catheter was filled with the solution. The open distal end was placed in the container mounted on a Kern CFS 3 k-5 scale (KERN & Sohn GmbH, Balingen, Germany). The catheter was fixed above the scale. A high-pressure inflation device (SIS Medical, Frauenfeld, Switzerland) for PTCA balloon catheters was attached to the hypotube and filled with 7 ml of the contrast media–saline solution. A vacuum was generated with the inflation device. The weight of the contrast media–saline solution was recorded every 3 s with the software BalanceConnection (KERN & Sohn GmbH, Balingen, Germany). The deflation rate [µL/s] was defined by the slope of the weight–time curve. Finally, the deflation time for a balloon with an OD of 3 mm and a length of 20 mm (assumed Volumen: 0.137 ml) was calculated.

## Figures and Tables

**Fig. 13:**
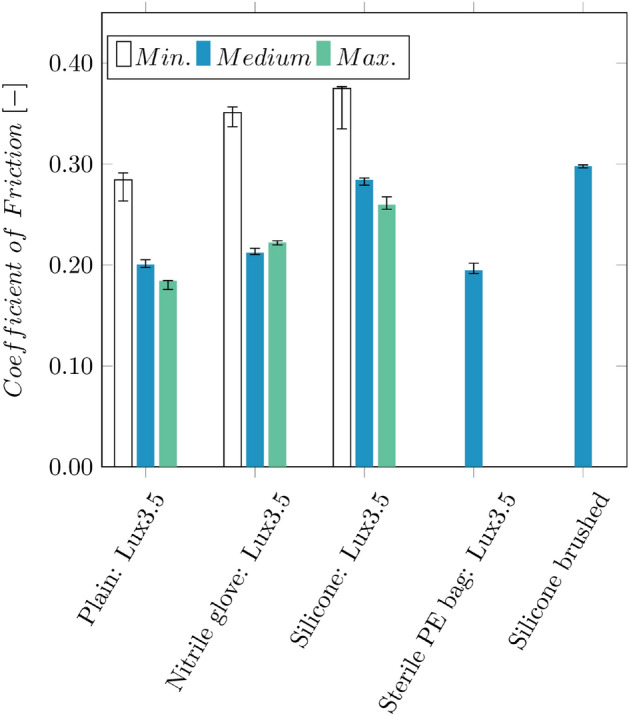
Comparison of the frictional coefficients between the hypotube of a Pantera Lux 3.5 DEB between the 3D-printed part (plain), nitrile rubber from gloves, silicone, and a sterile PE bag

**Fig. 14:**
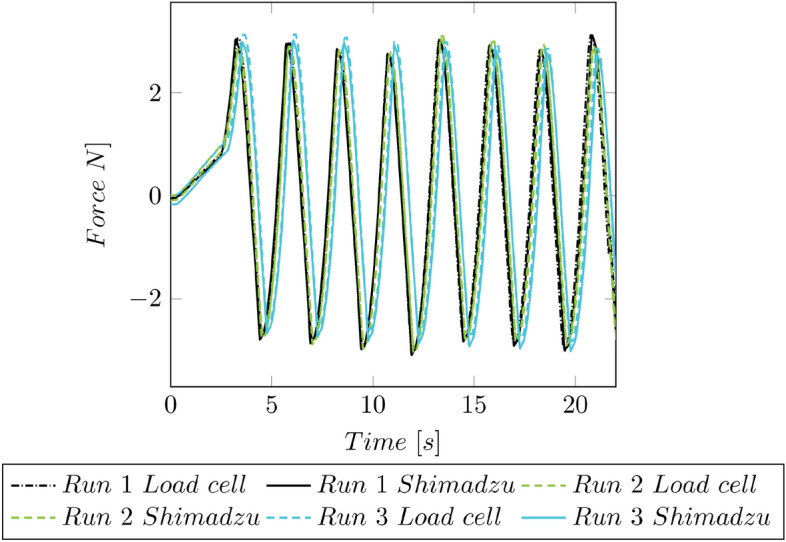
Force validation of the CFMD. The forces of the CFMD are compared to the forces of the universal testing machine Shimadzu AGS-X 10 kN

**Fig. 15:**
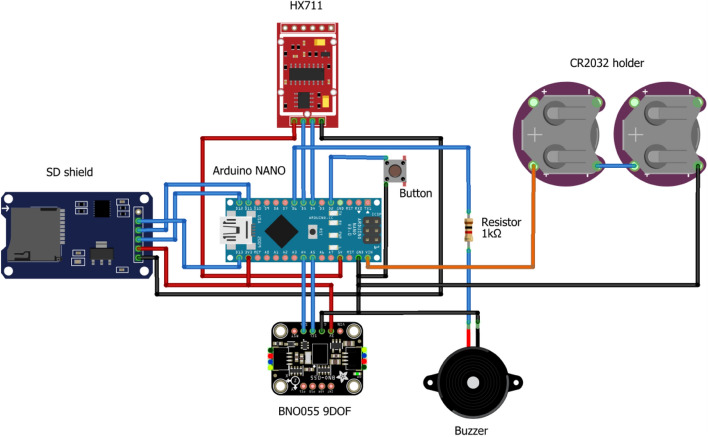
Wiring schematic of the used electronic components inside the CFMD

**Fig. 16:**
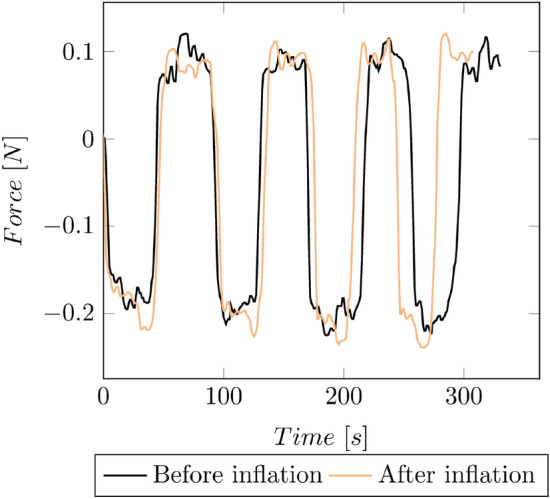
Reproducibility of a manual catheter measurement based on multiple push and pull cycles

## Data Availability

The datasets used and/or analyzed during the current study are available from the corresponding author upon reasonable request.

## Appendix

See Figs. 10, 11, 12, 13, 14, 15, 16

Creeping of the balloon in longitudinal direction after reaching the RBP:

The measured lengths of the various catheter sections (transition length, GW length, Stiffening wire length) and dimensions of the tapered stiffening wire:

Compliance chart and balloon growth given by the manufacturer:

### Development of the CFMD

The developed CFMD device consists of 3D-printed components (cf. Fig. 8). Due to the design, the handle can be carried with one hand. Forces (0–5 N) on a guidewire or a catheter with a minimum of 1 and a maximum of 6 French can be measured. Using a leg spring, the tool to be measured is fixed in the clamping unit. The spring can generate a minimum, medium, and maximum clamping force of 4.8, 15.5, and 24.9 N. Once the catheter is clamped, it can be pushed or pulled. Required forces for the movement will be recorded.

The contact surface between the clamps and the catheter is coated with nitrile rubber to increase friction and prevent catheter slippage. Figure 13 illustrates a comparison between various coatings and clamping forces. Different application methods, such as bonding and brushing, were compared for silicone. It is observed that using a nitrile glove increases friction compared to a plain surface. Furthermore, it was preferred compared to silicone due to the wear-off.

The clamping unit was tested for a catheter diameter between 2 and 6 French. These tests’ maximal achieved retention force was 4.76 N ± 0.30 N. To measure the force, a prefabricated force transducer using a Mini Load Cell with 500 g (SparkFun Electronics, Boulder, CO, USA), which indicates the forces with an accuracy smaller than ± 0.01 N. The force measurement was validated by attaching the load cell to the universal testing machine Shimadzu AGS-X 10 kN (Shimadzu Corporation, Kyoto, Japan) and applying an oscillating force over time. The forces recorded by the Shimadzu by a 200 N load cell (accuracy class: 0.5%) were then compared to the ones from the hand-held device (cf. Fig. 14).

In Fig. 15, the wiring schematic of the CFMD is shown. Inside the CFMD, a BNO055 (Robert Bosch AG, Gerlingen-Schillerhöhe, Germany) orientation sensor can record the device’s orientation in time. The data are stored on a 16 GB SD card, controlled by a battery-powered Arduino Nano (Arduino, Sommerv ille, MA; USA). An Arduino HX711 amplifies the load cell. Interaction with the device is enabled via a push button. A buzzer gives acoustic feedback about the status of the device.

For estimating the reproducibility, multiple push and pull cycles have been performed on one catheter (cf. Fig. 16).

## Abbreviations

CFMD: Catheter force measuring device
CP: Crossing profile
GC: Guide catheter
GW: Guide wire
IS: Inner shaft
IQR: Inter quartile range
LE: Lesion entry
NC: Non-compliant
OD: Outer diameter
OS: Outer shaft
PCI: Percutaneous coronary intervention
PEBAX: Low-density copolymer polyether block amide
PET: Polyethylene terephthalate
PTCA: Percutaneous transluminal coronary angioplasty
PVC: Polyvinyl chloride
RBP: Rated burst pressure
RX: Rapid exchange
SC: Semi-compliant
WT: Wall-thickness
